# Supplementary material for: Comprehensive Histopathology Imaging in Pancreatic Biopsies: High Definition Infrared Imaging with Machine Learning Approach
Source: Int J Biol Sci. 2023 Jun 19;19(10):3200–8. doi: 10.7150/ijbs.83068 (PMC10321284; doi:10.7150/ijbs.83068)
Supplement: Supplementary file 1 — Supplementary information and figures. [file ijbsv19p3200s1.pdf]

**Supplementary Materials to Comprehensive Histopathology Imaging in Pancreatic Biopsies: High Definition Infrared Imaging with Machine Learning Approach**

Danuta Liberda[1,2#], Paulina Koziol[2,3#], Tomasz P. Wrobel[2*]

[1] Jagiellonian University, Doctoral School of Exact and Natural Sciences, Prof. St. Łojasiewicza 11, PL30348, Cracow, Poland

[2] Solaris National Synchrotron Radiation Centre, Jagiellonian University, Czerwone Maki 98, 30-392
Krakow, Poland.
[3] Institute of Physics, Jagiellonian University, Lojasiewicza 11, 30-348 Krakow, Poland

*email: tomek.wrobel@uj.edu.pl
# equal contribution
Preprint available at: https://www.medrxiv.org/content/10.1101/2022.07.01.22277130v2

## Experimental details

In order to collect experimental data for multitude of samples within reasonable time frame, optimizations of measurement pipeline were necessary. Raw interferograms were Fourier-Transformed and stitched using separate computer, making no impact on measurement time. Combination of this approach and regular detector pumping allowed continuous measurement of large areas for 10-13.5 hours (without liquid nitrogen refill). Nonetheless, measurements of large areas come with the risk of defocusing due to sample tilt. This issue was overcome by implementation of in home-written scripts for focusing modality, which resulted in high quality data. Optimization and customization of measurement routines elevated the scanning time of single TMA (20x16 mm in size) to 2.5 days for SD and 2.5 weeks for HD.

## Model validation

Two different ways for ROC and AUC calculation were applied during different stages of final model's creation. Pixel level ROC statistic was used during an iterative process of classifier training/tuning to assess its ability for a successful single pixel classification. ROC curve for each class was calculated based on biased two class model (specific class versus five other classes merged into one). Initial probability ratio of such models was always 1:5, due to classes' size imbalance and the thresholds used for ROC calculations are based on trees voting. This approach is very different from conventional way applied here to evaluated final model. Core level and patient level ROC statistic are based on summation of prediction pixels from each tissue core or patient, respectively. A statistical analysis in the form of confidence intervals was applied to obtained values of AUC. Standard error for AUC is evaluated as:

$$SE(AUC) = \sqrt{\frac{AUC(1-AUC) + (n_1 - 1)(Q_1 - AUC^2) + (n_0 - 1)(Q_2 - AUC^2)}{n_0 n_1}},$$

where $n_0$ is the number of samples for positive class, $n_1$ is the number of other samples and:

$$Q_1 = \frac{AUC}{2 - AUC}, \qquad Q_2 = \frac{2AUC^2}{1 + AUC}.$$

In general, histopathological model creation is tedious and iterative process. It consists of many rounds of annotations corrections and optimizations. In case of this study, hundredths of models were created before achieving satisfactory results.

**Table S1.** Description of patients with Tissue Micro Arrays (TMA) location and cores represented with BIOMAX Inc. notation (Letter – TMA row, Number – TMA column).

| Patient number | PA2081b | PA2072a | PA1002a | PA961e | BBS14011 | BIC14011a |
|---|---|---|---|---|---|---|
| 1 | A1,A2 | - | - | B3 | - | - |
| 2 | A3,A4 | - | A4 | D10 | - | - |
| 3 | A5,A6 | - | - | - | - | - |
| 4 | A7,A8 | - | F4,F9 | E7 | - | - |
| 5 | A9,A10,I5,I6 | - | A2,A7 | - | - | - |
| 6 | A11,A12 | B3,B4,B5 | - | - | - | - |
| 7 | A13,A14 | B12,B13,B14 | C3,C8 | A3 | - | - |
| 8 | A15,A16 | C8,C9,C10 | C4,C9 | - | - | - |
| 9 | B1,B2 | - | A3 | B10 | - | - |
| 10 | B3,B4 | - | - | C2 | - | - |
| 11 | B5,B6 | - | - | - | - | - |
| 12 | B7,B8 | - | H2,H7 | F12 | - | - |
| 13 | B9,B10 | G7,G8,G9 | B5,B10 | - | - | - |
| 14 | B11,B12 | - | - | - | - | - |
| 15 | B13,B14 | E12,E13,E14 | E5, E10 | - | - | - |
| 16 | B15,B16 | - | - | - | - | - |
| 17 | C1,C2 | E15,E16,F1 | C2,C7 | C12 | - | - |
| 18 | C3,C4 | - | - | - | - | - |
| 19 | C5,C6 | - | - | - | - | - |
| 20 | C7,C8 | G16,H1,H2 | - | C8 | - | - |
| 21 | C9,C10 | I5,I6,I7 | E3,F8 | E6 | - | - |
| 22 | C11,C12 | D13,D14,D15 | - | B9 | - | - |
| 23 | C13,C14 | A16,B1,B2 | - | - | - | F1,F2 |
| 24 | C15,C16 | - | B3,B8 | C10 | - | - |
| 25 | D1,D2 | - | B1,B6 | A6 | - | - |
| 26 | D3,D4 | - | - | - | - | - |
| 27 | D5,D6 | H9,H10,H11 | - | C1 | - | - |
| 28 | D7,D8 | - | - | - | - | - |
| 29 | D9,D10 | - | - | G1 | - | - |
| 30 | D11,D12 | - | - | - | - | - |
| 31 | D13,D14 | G1,G2,G3 | E2,E7 | G2 | - | - |
| 32 | D15,D16 | - | - | - | - | - |
| 33 | E1,E2 | - | - | - | - | - |
| 34 | E3,E4 | - | - | - | - | - |
| 35 | E5,E6 | - | - | - | - | - |
| 36 | E7,E8 | - | - | G3 | - | - |
| 37 | E9,E10 | - | G4,G9 | - | - | - |
| 38 | E11,E12 | J7,J8,J9 | G5,G10 | - | - | - |
| 39 | E13,E14 | - | - | G5 | - | - |
| 40 | E15,E16 | - | - | - | - | - |
| 41 | F1,F2 | - | H1,H6 | - | - | - |
| 42 | F3,F4 | C2,C3,C4 | - | - | - | - |
| 43 | F5,F6 | - | - | - | - | - |
| 44 | F7,F8 | - | - | G11 | - | - |
| 45 | F10 | - | - | G10 | - | - |

| | | | | | | |
|---|---|---|---|---|---|---|
| 46 | - | - | - | - | - | - |
| 47 | - | - | - | - | - | - |
| 48 | - | - | - | - | - | - |
| 49 | - | - | - | - | - | - |
| 50 | - | - | - | - | - | - |
| 51 | - | - | - | - | - | - |
| 52 | - | - | - | - | - | - |
| 53 | G9,G10 | - | - | - | - | - |
| 54 | G11,G12 | - | - | - | - | - |
| 55 | G13,G14 | - | - | - | - | - |
| 56 | G15,G16 | - | - | - | - | - |
| 57 | H1,H2 | - | - | - | - | - |
| 58 | H3,H4 | - | - | - | - | - |
| 59 | H5,H6 | - | - | - | - | - |
| 60 | H7,H8 | - | - | - | - | - |
| 61 | H9,H10 | - | - | - | - | - |
| 62 | H11,H12 | - | - | - | - | - |
| 63 | H13,H14 | - | - | - | - | - |
| 64 | H15,H16 | - | - | - | - | - |
| 65 | I1,I2 | - | - | - | - | - |
| 66 | I3,I4 | - | - | - | - | - |
| 67 | I7,I8 | - | - | - | - | - |
| 68 | I9,I10 | - | - | - | - | - |
| 69 | I11 | - | - | - | - | - |
| 70 | I13,I14 | - | - | - | - | - |
| 71 | I15,I16 | - | - | - | - | - |
| 72 | J1,J2 | - | - | - | - | - |
| 73 | J3,J4 | - | - | - | - | - |
| 74 | J5,J6 | - | - | - | - | - |
| 75 | - | - | - | - | - | - |
| 76 | J9,J10 | - | - | - | - | - |
| 77 | J11,J12 | - | - | - | - | - |
| 78 | J13,J14 | - | - | - | - | - |
| 79 | J15,J16 | - | - | - | - | - |
| 80 | K1,K2 | - | - | - | - | - |
| 81 | K3,K4 | - | - | - | - | - |
| 82 | K5,K6 | - | - | - | - | - |
| 83 | K7,K8 | - | - | - | - | - |
| 84 | K9,K10 | - | - | - | - | - |
| 85 | K11,K12 | - | - | C9 | - | - |
| 86 | K13,K14 | A13,A14,A15 | - | B2 | - | - |
| 87 | K15,K16 | - | - | - | - | - |
| 88 | L1,L2 | - | - | - | - | - |
| 89 | L3,L4 | - | - | - | - | - |
| 90 | L5,L6 | - | - | - | - | - |
| 91 | L7,L8 | - | - | - | - | - |
| 92 | L9,L10 | - | - | - | A3 | - |
| 93 | L11,L12 | - | - | - | - | - |
| 94 | L13,L14 | - | - | - | - | - |
| 95 | L15,L15 | M4,M5,M6 | J1,J6 | - | - | - |
| 96 | - | L5,L6,L7 | J4,J9 | - | - | - |
| 97 | M3,M4 | - | I10 | - | - | - |
| 98 | M5,M6 | - | I9 | - | - | - |
| 99 | M7,M8 | - | J3,J8 | - | - | - |
| 100 | M9,M10 | M3 | I6 | - | - | - |
| 101 | M11,M12 | L11,L12,L13 | J2,J7 | - | - | - |
| 102 | M13,M14 | - | I8 | - | - | - |
| 103 | M15,M16 | M10,M11,M12 | I7 | - | - | - |

| | | | | | | |
|---|---|---|---|---|---|---|
| 104 | - | A1,A2,A3 | - | - | - | - |
| 105 | - | A4,A5,A6 | - | - | - | - |
| 106 | - | A7,A8 | - | - | - | - |
| 107 | - | A10,A11,A12 | - | B4 | - | - |
| 108 | - | B6,B7,B8 | - | - | - | - |
| 109 | - | B9,B10,B11 | - | - | - | - |
| 110 | - | B15,B16,C1 | - | B11 | - | - |
| 111 | - | C5,C6,C7 | - | - | - | - |
| 112 | - | C11,C12,C13 | - | - | - | - |
| 113 | - | C14,C15,C16 | - | B8 | - | - |
| 114 | - | D1,D2,D3 | - | E9 | - | - |
| 115 | - | D4,D5,D6 | - | - | - | - |
| 116 | - | D7,D8,D9 | - | - | - | - |
| 117 | - | D10,D11,D12 | - | D8 | - | - |
| 118 | - | D16,E1,E2 | D4,D9 | C5 | - | - |
| 119 | - | E3,E4,E5 | - | C3 | - | F5,F6 |
| 120 | - | E6,E7,E8 | D2,D7 | D6 | - | - |
| 121 | - | E9,E10,E11 | - | - | - | - |
| 122 | - | F2,F3,F4 | - | E2 | - | - |
| 123 | - | F5,F6,F7 | - | - | - | - |
| 124 | - | F8,F9,F10 | C1,C6 | - | - | - |
| 125 | - | F11,F12,F13 | B4,B9 | C4 | - | - |
| 126 | - | F14,F15,F16 | - | D1 | - | - |
| 127 | - | G4,G5,G6 | - | - | - | - |
| 128 | - | G10,G11,G12 | - | - | - | - |
| 129 | - | G13,G14,G15 | - | - | - | - |
| 130 | - | H5 | - | - | - | - |
| 131 | - | H6,H7,H8 | - | - | - | - |
| 132 | - | H12,H13,H14 | H3,H8 | - | - | - |
| 133 | - | H15,H16,I1 | - | - | - | - |
| 134 | - | I2,I3,I4 | - | - | - | - |
| 135 | - | I8,I9,10 | - | - | - | F3,F4 |
| 136 | - | I11,I12,I13 | - | G6 | - | - |
| 137 | - | I14,I15,I16 | - | - | - | - |
| 138 | - | J1,J2,J3 | - | F3 | - | - |
| 139 | - | J4,J5,J6 | G3,G8 | E11 | - | - |
| 140 | - | J10,J11,J12 | C5,C10 | F10 | - | - |
| 141 | - | J13,J14,J15 | F5,F10 | - | - | - |
| 142 | - | J16,K1,K2 | - | - | - | F7,F8 |
| 143 | - | K3,K4,K5 | H5,H10 | F11 | - | - |
| 144 | - | K6,K7,K8 | - | F7 | - | - |
| 145 | - | K9,K10,K11 | - | - | - | - |
| 146 | - | K12,K13,K14 | - | E3 | - | - |
| 147 | - | K15,K16,L1 | F1,F6 | D2 | - | - |
| 148 | - | L2,L3,L4 | - | - | E6 | - |
| 149 | - | L8,L9,L10 | - | H12 | - | - |
| 150 | - | L14,L15,L16 | - | - | - | - |
| 151 | - | M7,M8,M9 | - | - | - | - |
| 152 | - | M13,M14,M15 | - | D3 | - | - |
| 153 | - | - | A1,B7 | - | - | - |
| 154 | - | - | A5,D1 | - | - | - |
| 155 | - | - | A6,D3 | E5 | - | - |
| 156 | - | - | A10,D5 | - | - | - |
| 157 | - | - | B2,D6 | - | - | - |
| 158 | - | - | D8,E6 | E4 | - | - |
| 159 | - | - | D10,E8 | E1 | - | - |
| 160 | - | - | E1,E9 | - | - | - |
| 161 | - | - | E3,E2 | - | - | - |

| | | | | | | |
|---|---|---|---|---|---|---|
| 162 | - | - | E4,F7 | - | - | - |
| 163 | - | - | G1 | F1 | - | - |
| 164 | - | - | G2,J5 | - | - | - |
| 165 | - | - | G6,J10 | F8 | - | - |
| 166 | - | - | G7 | F6 | - | - |
| 167 | - | - | H4 | - | - | - |
| 168 | - | - | - | A1 | - | - |
| 169 | - | - | - | A2 | - | - |
| 170 | - | - | - | A4 | - | - |
| 171 | - | - | - | A5 | - | - |
| 172 | - | - | - | A7 | - | - |
| 173 | - | - | - | A8 | - | - |
| 174 | - | - | - | A10 | - | - |
| 175 | - | - | - | A11 | - | - |
| 176 | - | - | - | A12 | - | - |
| 177 | - | - | - | B1 | - | - |
| 178 | - | - | - | B5 | - | - |
| 179 | - | - | - | B6 | - | - |
| 180 | - | - | - | B7 | - | - |
| 181 | - | - | - | B12 | - | - |
| 182 | - | - | - | C6 | - | - |
| 183 | - | - | - | C7 | - | - |
| 184 | - | - | - | - | - | - |
| 185 | - | - | - | D4 | D2 | - |
| 186 | - | - | - | D5 | - | - |
| 187 | - | - | - | D7 | - | - |
| 188 | - | - | - | D9 | - | - |
| 189 | - | - | - | D11 | - | - |
| 190 | - | - | - | D12 | - | - |
| 191 | - | - | - | - | - | - |
| 192 | - | - | - | E10 | - | - |
| 193 | - | - | - | F2 | - | - |
| 194 | - | - | - | F4 | - | - |
| 195 | - | - | - | F5 | - | - |
| 196 | - | - | - | F9 | - | - |
| 197 | - | - | - | G4 | - | - |
| 198 | - | - | - | - | - | - |
| 199 | - | - | - | G8 | - | - |
| 200 | - | - | - | G9 | - | - |
| 201 | - | - | - | G12 | - | - |
| 202 | - | - | - | H1 | - | - |
| 203 | - | - | - | H2 | - | - |
| 204 | - | - | - | H3 | - | - |
| 205 | - | - | - | H4 | - | - |
| 206 | - | - | - | H5 | - | - |
| 207 | - | - | - | H6 | - | - |
| 208 | - | - | - | - | - | - |
| 209 | - | - | - | - | - | - |
| 210 | - | - | - | - | A1 | - |
| 211 | - | - | - | - | A2 | - |
| 212 | - | - | - | - | A4 | B7,B8 |
| 213 | - | - | - | - | A5 | - |
| 214 | - | - | - | - | A6 | - |
| 215 | - | - | - | - | A7 | - |
| 216 | - | - | - | - | A8 | C3,C4 |
| 217 | - | - | - | - | B1 | - |
| 218 | - | - | - | - | B2 | - |
| 219 | - | - | - | - | B3 | - |

| | | | | | | |
|---|---|---|---|---|---|---|
| 220 | - | - | - | - | B4 | - |
| 221 | - | - | - | - | B5 | - |
| 222 | - | - | - | - | B6 | - |
| 223 | - | - | - | - | B7 | B1,B2 |
| 224 | - | - | - | - | B8 | - |
| 225 | - | - | - | - | C1 | - |
| 226 | - | - | - | - | C2 | A3,A4 |
| 227 | - | - | - | - | C3 | - |
| 228 | - | - | - | - | C4 | - |
| 229 | - | - | - | - | C5 | - |
| 230 | - | - | - | - | C6 | - |
| 231 | - | - | - | - | - | - |
| 232 | - | - | - | - | C8 | - |
| 233 | - | - | - | - | D1 | - |
| 234 | - | - | - | - | D3 | - |
| 235 | - | - | - | - | D4 | - |
| 236 | - | - | - | - | D5 | - |
| 237 | - | - | - | - | D6 | - |
| 238 | - | - | - | - | D7 | A5,A6 |
| 239 | - | - | - | - | D8 | B3,B4 |
| 240 | - | - | - | - | E1 | - |
| 241 | - | - | - | - | E2 | - |
| 242 | - | - | - | - | E3 | - |
| 243 | - | - | - | - | E4 | - |
| 244 | - | - | - | - | E5 | - |
| 245 | - | - | - | - | E7 | - |
| 246 | - | - | - | - | E8 | - |
| 247 | - | - | - | - | F1 | - |
| 248 | - | - | - | - | F2 | - |
| 249 | - | - | - | - | F3 | B5,B6 |
| 250 | - | - | - | - | F4 | E3,E4 |
| 251 | - | - | - | - | F5 | - |
| 252 | - | - | - | - | F6 | C1,C2 |
| 253 | - | - | - | - | F7 | E7,E8 |
| 254 | - | - | - | - | F8 | - |
| 255 | - | - | - | - | - | A1,A2 |
| 256 | - | - | - | - | - | A7,A8 |
| 257 | - | - | - | - | - | C5,C6 |
| 258 | - | - | - | - | - | C7,C8 |
| 259 | - | - | - | - | - | D1,D2 |
| 260 | - | - | - | - | - | D3,D4 |
| 261 | - | - | - | - | - | D5,D6 |
| 262 | - | - | - | - | - | D7,D8 |
| 263 | - | - | - | - | - | E1,E2 |
| 264 | - | - | - | - | - | E5,E6 |
| **Total cores number** | | | | | | |
| 663 | | | | | | |
| **Total patients number** | | | | | | |
| 250 | | | | | | |

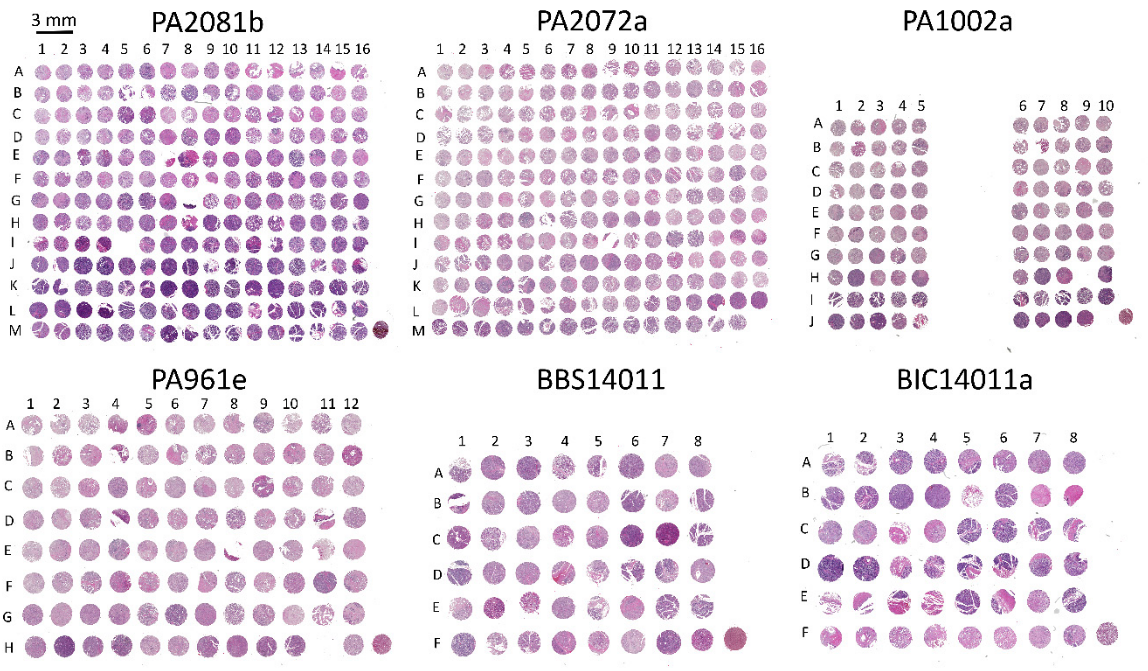


**Figure S1.** H&E images of pancreatic Micro Tissue Arrays used in this study. Cores in each matrix are numbered according to BIOMAX Inc. notation.

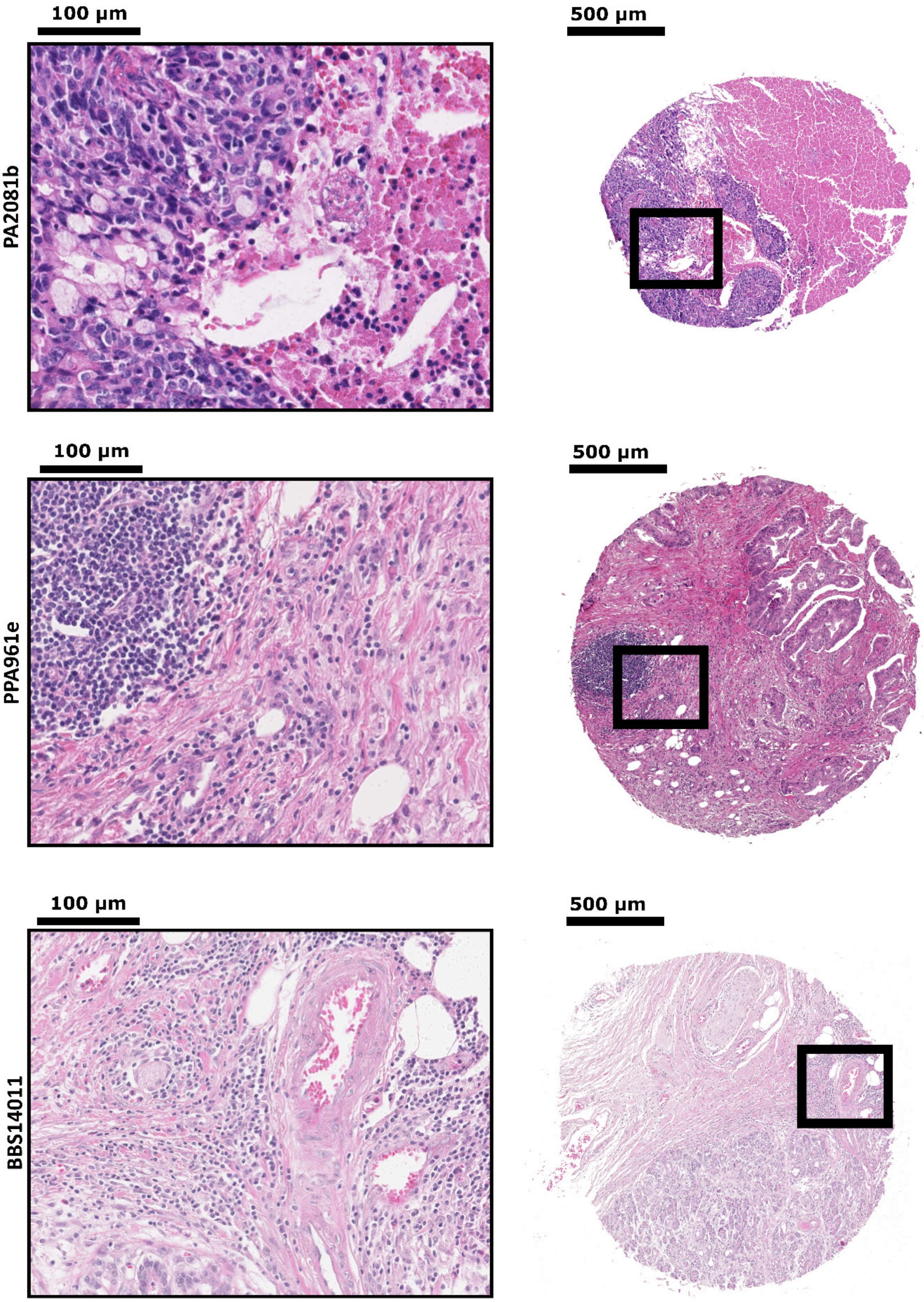


**Figure S2.** H&E images of zoomed biopsies (from TMAs PA2081b, PA961e, BBS14011) shown in Figures 2 and 4.

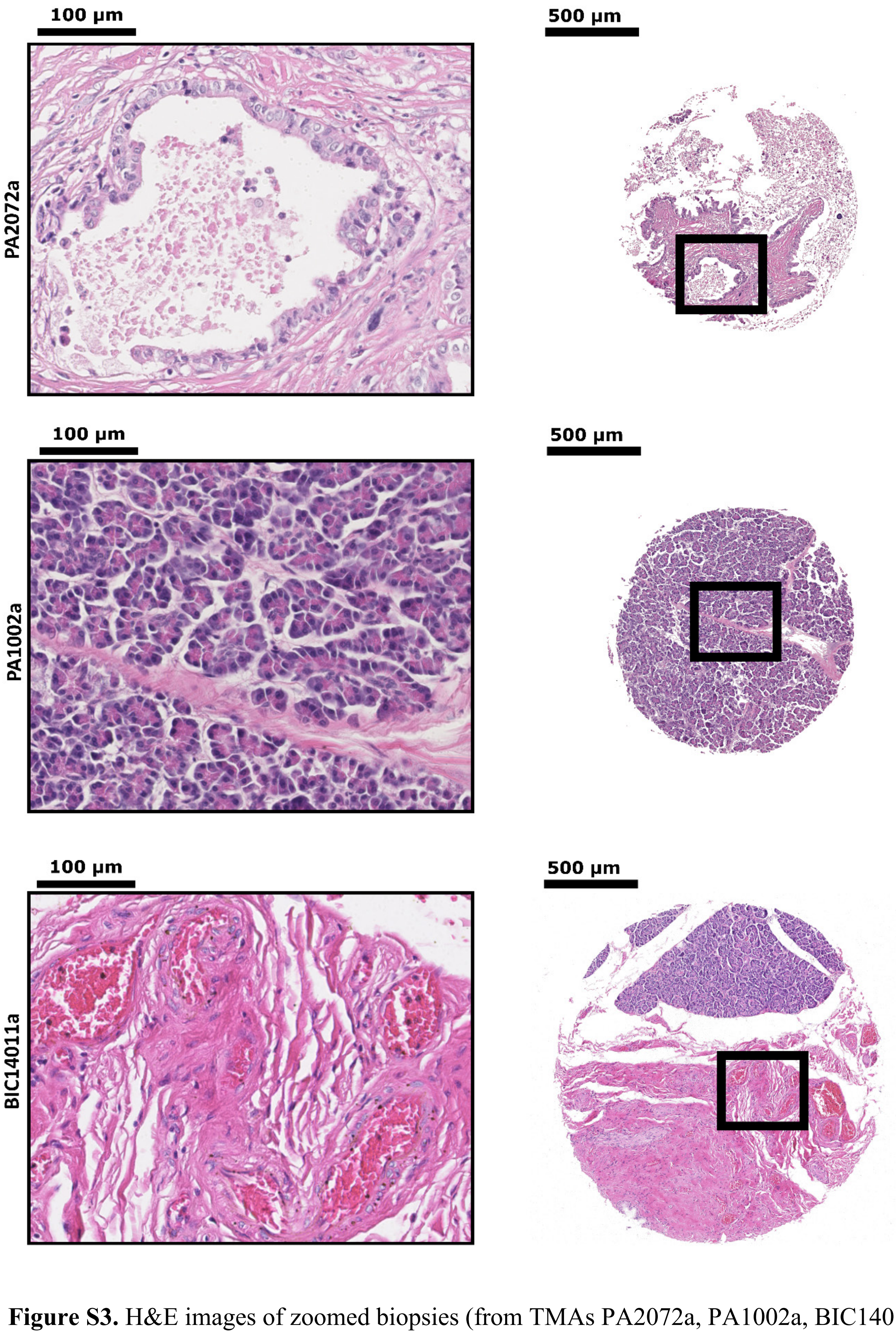


**Figure S3.** H&E images of zoomed biopsies (from TMAs PA2072a, PA1002a, BIC14011a) shown in Figures 2 and 4.

## Spectral regions used for biochemical information extraction

**Table S2.** List of spectral regions used for biochemical information extraction. Additional region of 1586-1705 $cm^{-1}$ (Amide I), was defined for normalization purpose.

| **No.** | **Band range [$cm^{-1}$]** | |
|---|---|---|
| 1 | 940 | 980 |
| 2 | 1015 | 1040 |
| 3 | 980 | 1050 |
| 4 | 1040 | 1070 |
| 5 | 980 | 1100 |
| 6 | 1050 | 1100 |
| 7 | 980 | 1140 |
| 8 | 1050 | 1140 |
| 9 | 1100 | 1140 |
| 10 | 1010 | 1155 |
| 11 | 1160 | 1180 |
| 12 | 1140 | 1180 |
| 13 | 1190 | 1210 |
| 14 | 1180 | 1210 |
| 15 | 1210 | 1280 |
| 16 | 1190 | 1290 |
| 17 | 1180 | 1290 |
| 18 | 1210 | 1290 |
| 19 | 1263 | 1296 |
| 20 | 1295 | 1320 |
| 21 | 1295 | 1325 |
| 22 | 1325 | 1346 |
| 23 | 1340 | 1360 |
| 24 | 1360 | 1390 |
| 25 | 1346 | 1420 |
| 26 | 1360 | 1425 |
| 27 | 1420 | 1478 |
| 28 | 1425 | 1480 |
| 29 | 1478 | 1586 |
| 30 | 1490 | 1590 |
| 31 | 1710 | 1760 |
| 32 | 2825 | 2860 |
| 33 | 2800 | 2880 |
| 34 | 2880 | 2940 |
| 35 | 2940 | 2980 |
| 36 | 2800 | 3025 |
| 37 | 3000 | 3025 |
| 38 | 2995 | 3095 |
| 39 | 3025 | 3100 |
| 40 | 2995 | 3570 |
| 41 | 3100 | 3600 |

## Validation description

**Cancer class** includes patient annotations: Malignant. **Inflammation class** includes patient annotation Inflammation. **Healthy class** includes patient annotation: Normal, NAT, PanIN.

**Table S3.** FT-IR patient annotations to folds in cross validation and test sets for Random Forest classification. Patients remaining in each set were assigned to a given model set.

| **validation type_fold** | **Patient annotation** | | |
|---|---|---|---|
| | **Cancer** | **Inflammation** | **Healthy** |
| Cross_validation_set_1 | 24, 28, 29, 35, 39, 42, 44, 86, 106, 113, 114, 120, 123, 124, 126, 128, 132, 134, 139, 153, 155, 156, 162, 165, 177, 192, 193, 196, 200 | 185, 214, 220, 223, 239, 240, 241, 242, 243, 244, 245, 247, 248 | 87, 91, 95, 97, 102, 149, 262, 263 |
| Cross_validation _set_2 | 2, 5, 11, 13, 15, 16, 19, 25, 36, 37, 55, 58, 59, 61, 62, 110, 121, 127, 130, 138, 141, 144, 154, 167, 171, 172, 179, 183, 186, 187, 207 | 64, 72, 92, 217, 219, 222, 224, 225, 228, 229, 235, 260, 261 | 73, 74, 78, 88, 90, 93, 100 |

| | | | |
|---|---|---|---|
| Cross_validation _set_3 | 3, 7, 9, 12, 17, 22, 26, 30, 31, 32, 34, 40, 43, 53, 107, 108, 109, 111, 118, 142, 147, 157, 158, 160, 169, 176, 178, 180, 190, 194, 203 | 65, 69, 70, 215, 226, 227, 230, 232, 246, 249, 253, 258, 259 | 79, 84, 89, 99, 103, 150, 151 |
| Cross_validation _set_4 | 4, 8, 14, 18, 21, 23, 41, 45, 56, 60, 104, 112, 116, 133, 137, 143, 159, 163, 164, 168, 170, 188, 189, 197, 199, 201, 202, 205, 206 | 66, 67, 148, 211, 212, 213, 221, 233, 234, 236, 251, 252, 255 | 76, 77, 82, 83, 85, 94, 96 |
| External_test_set | 1, 6, 10, 20, 27, 33, 38, 54, 57, 105, 115, 117, 119, 122, 125, 129, 131, 135, 136, 140, 145, 146, 161, 166, 173, 174, 175, 181, 182, 195, 204 | 63, 68, 71, 210, 216, 218, 237, 238, 250, 254, 256, 257, 264 | 80, 81, 98, 101, 152 |
